# Professional Secrecy

**Published:** 1899-03-18

**Authors:** 


### The Hospital

A Journal of

The Medical Sciences and Hospital Administration.

Vol. XXV.—No. 651.] Edited by Sir Henry Burdett, K.C.B., and Solomon C. Smith, M.D., M.R.C.P. [March 18, 1899.

### Professional Secrecy.

At the meeting of the Executive Committee of the General Medical Council on the 27th ult. a letter was read from the Home Office, covering an application from the Russian Ambassador, in which he requested to be furnished, for the use of his Government, with information as to the law of this country in regard to the professional secrecy of medical practitioners. By direction of the President, the subject had been referred to Mr. Muir Mackenzie, whose opinion was before the Committee, and was ordered to be sent to the Home Secretary. It contains what may be regarded as an authoritative statement of the law upon a question not altogether free from difficulties; and it is, therefore, desirable to place its most important features upon record.

Mr. Muir Mackenzie divides the question submitted to him into two parts: first, as to whether a medical man can be *made* to divulge professional confidences in a court of law; secondly, as to whether he may in any circumstances be *permitted* to divulge professional confidences in the intercourse of private life. With regard to the first of these, he cites a number of judicial authorities, from Lord Mansfield downwards, to show that, as a matter of settled law, a medical practitioner has no privilege of refusing to answer, as a witness, concerning matters which have been communicated to him professionally. "If a surgeon was voluntarily to reveal these secrets," said Lord Mansfield in the Duchess of Kingston's case, "to be sure he would be guilty of a breach of honour and of great indiscretion; but to give that information in a court of justice, which, by the law of the land he is bound to do, will never be imputed to him as any indiscretion whatever." Mr. Justice Buller, somewhat later, after referring to a lawyer's right of privilege, observed that it was not very easy to discover why a like privilege has been refused to others, especially to medical advisers; and Mr. Justice Park, in the case of a woman indicted for the murder of her child, overruled the objection that a surgeon who was called as a witness for the prosecution had been attending the prisoner professionally, saying, "That is no sufficient reason to prevent a disclosure for the purposes of justice." A like decision was cited from Chief Justice Best; and, on the whole, it is clear that a medical man not only may, but must, if necessary, violate professional confidences when answering questions material to an issue in a court of law. It should be remembered, however, that, especially in cross-examination, counsel might put a question the reply to which would involve a breach of confidence, and which, in the opinion of the witness, might not be material to the issue. In any such case the proper course would be to appeal to the Judge, and to be absolutely guided by his decision.

With regard to the second question, Mr. Muir Mackenzie was not able to speak in an equally decided manner, although it has been judicially decided in the Scotch Court of Session that "secrecy is an essential condition of the contract between a medical man and his employers, and breach of secrecy affords a relevant ground for an action of damages." He quotes Mr. Justice Hawkins, who said, in his summing up in the case of Kitson v. Playfair, that "the medical profession might, no doubt, discuss among themselves rules for their own guidance, but that they had not power to impose these rules upon the public," and who adversely criticised the view that a medical man was necessarily called upon to communicate with public authorities because he supposed that a patient had intended to commit a crime. "If the doctor were called in to attend a woman needing physical aid," his Lordship doubted very much whether he would be justified in going to the police and saying, "I have been attending a woman who has been trying to procure an abortion." "That would be monstrous cruelty." Mr. Muir Mackenzie's final conclusion is that, on this question a medical man is in no more favoured position than anyone else, and, further, that circumstances, which according to the custom of the medical profession might be deemed to exonerate him from the imputation of improper violation of secrecy, might nevertheless in a court of law be deemed an insufficient justification. The only safe rule, we believe, is to decline to make any communication to a third person about a patient, unless the consent of the patient has first been given in writing, or in the presence of a credible witness. We know of one instance in which a medical man had to pay damages for communicating to a professedly sympathetic inquirer an opinion as to the state of a patient, whose intended marriage to an heiress was broken off in consequence of the information thus imparted; and a medical man employed by the mistress of a household has before now suffered in like manner from communicating his knowledge or suspicion of the pregnancy of a servant. It is safe to speak freely to a parent about a child who is under age, but, in all other circumstances, it is hardly possible to be too cautious.

